# Genetic stratification of depression by neuroticism: revisiting a diagnostic tradition

**DOI:** 10.1017/S0033291719002629

**Published:** 2020-11

**Authors:** Mark J. Adams, David M. Howard, Michelle Luciano, Toni-Kim Clarke, Gail Davies, W. David Hill, 23andMe Research Team, Major Depressive Disorder Working Group of the Psychiatric Genomics Consortium, Daniel Smith, Ian J. Deary, David J. Porteous, Andrew M. McIntosh

**Affiliations:** 1 Division of Psychiatry, University of Edinburgh, Royal Edinburgh Hospital, Edinburgh, UK; 2 Social, Genetic and Developmental Psychiatry, Institute of Psychiatry, Psychology and Neuroscience, King's College London, London, UK; 3 Centre for Cognitive Ageing and Cognitive Epidemiology, University of Edinburgh, Edinburgh, UK; 4 Department of Psychology, University of Edinburgh, Edinburgh, UK; 5 23andMe, Inc., Mountain View, CA, USA; 6 Institute of Health and Wellbeing, University of Glasgow, Glasgow, UK; 7 Centre for Genomic and Experimental Medicine, Institute of Genetics and Molecular Medicine, University of Edinburgh, Edinburgh, UK

**Keywords:** Diagnosis, genetic correlation, genome-wide association study, major depressive disorder, neuroticism

## Abstract

**Background:**

Major depressive disorder and neuroticism (Neu) share a large genetic basis. We sought to determine whether this shared basis could be decomposed to identify genetic factors that are specific to depression.

**Methods:**

We analysed summary statistics from genome-wide association studies (GWAS) of depression (from the Psychiatric Genomics Consortium, 23andMe and UK Biobank) and compared them with GWAS of Neu (from UK Biobank). First, we used a pairwise GWAS analysis to classify variants as associated with only depression, with only Neu or with both. Second, we estimated partial genetic correlations to test whether the depression's genetic link with other phenotypes was explained by shared overlap with Neu.

**Results:**

We found evidence that most genomic regions (25/37) associated with depression are likely to be shared with Neu. The overlapping common genetic variance of depression and Neu was genetically correlated primarily with psychiatric disorders. We found that the genetic contributions to depression, that were not shared with Neu, were positively correlated with metabolic phenotypes and cardiovascular disease, and negatively correlated with the personality trait conscientiousness. After removing shared genetic overlap with Neu, depression still had a specific association with schizophrenia, bipolar disorder, coronary artery disease and age of first birth. Independent of depression, Neu had specific genetic correlates in ulcerative colitis, pubertal growth, anorexia and education.

**Conclusion:**

Our findings demonstrate that, while genetic risk factors for depression are largely shared with Neu, there are also non-Neu-related features of depression that may be useful for further patient or phenotypic stratification.

## Introduction

Major depressive disorder (MDD) is a leading cause of morbidity worldwide, currently affecting approximately 4% of the world's population (World Health Organization, 2017). MDD is classified by the World Health Organisation and American Psychiatric Association according to its severity, its recurrence or chronicity, and the presence or absence of psychotic symptoms. This approach aims to maximise the reliability of MDD's diagnosis while being agnostic about its underlying aetiology until robust evidence of causal mechanisms can be used to stratify the condition.

Historically, depression had been classified on the basis of pre-existing emotional instability into ‘neurotic’ and ‘endogenous’ forms (Kendell, 1977). ‘Neurotic depression’ was diagnosed in the presence of pre-existing emotional instability and was reported to occur in younger individuals who more frequently expressed suicidal ideation. ‘Endogenous depression’, in contrast, was characterised by more frequent melancholic symptoms, including disrupted sleep, impaired appetite, diurnal variation of mood and impaired cognition. Individuals with endogenous depression were described as more responsive to antidepressant treatments (Fava *et al*., 2008).

The nosological status of neurotic and endogenous depression has, however, been very controversial (Parker, 2000). Some studies have found empirical support for the neurotic/endogenous division based upon self-report and clinical data (Zimmerman *et al*., 1986), whereas other studies found little empirical support for the distinction and noted that individuals with low mood responded to antidepressant treatments regardless of their subtype (Arnow *et al*., 2015).

The tendency towards emotional instability, or neuroticism (Neu), is a robustly and consistently replicated dimension of personality that is relatively stable over time (Eysenck, 1947). Neu features in the most widely accepted theory of personality structure, the Five Factor model, alongside openness to experience, extraversion, conscientiousness and agreeableness (Digman, 1990). Trait Neu (sometimes also labelled as emotionality or its opposite, emotional stability) is identified consistently and is composed of items reflecting low mood, stress sensitivity, irritability and emotional control (McCrae and Costa, 1987).

Twin, family and genomic studies have shown that population variation in Neu and liability to depression are conferred by both genetic and environmental risk factors (Sullivan *et al*., 2000; Keller *et al*., 2005; Lubke *et al*., 2012; Boraska *et al*., 2014; Zeng *et al*., 2016; Hill *et al*., 2018). While earlier studies (Major Depressive Disorder Working Group of the Psychiatric GWAS Consortium, 2013) had difficulty in discovering specific genetic variants for depression, possibly partly because of heterogeneity (Levinson *et al*., 2014), recent studies have identified genome-wide significantly associated loci either by focusing on more rigorously defined MDD phenotypes (CONVERGE Consortium, 2015) or by meta-analysing case–control samples with larger sample-size studies that use broader major depression (MD) phenotypes (Hyde *et al*., 2016; Direk *et al*., 2017; Howard *et al*., 2018, 2019; Wray *et al*., 2018; Amare *et al*., 2019). Results from genome-wide association studies (GWAS) of MD primarily implicate brain-expressed genes, developmental regulatory pathways and education and body mass index (BMI) as causal factors. Recent studies of Neu have also identified over 100 genome-wide significant loci (Luciano *et al*., 2017; Nagel *et al*., 2018), and likewise are associated with genes involved in neuronal genesis. More recent work examining the heterogeneity of Neu has identified many more loci that are specific to the traits that make-up Neu. Confirming earlier findings from biometric studies (Fanous *et al*., 2002; Kendler *et al*., 2006), these studies have shown that depression and Neu have a genetic correlation of between 0.45 and 0.7 and thus share a moderate to substantial proportion of their genetic architecture (Luciano *et al*., 2017). Since this genetic correlation is sizeable but still less than 1, it suggests that depression and Neu are either both unreliable measures of the same underlying genetic predisposition or it indicates that depression and Neu may not be identical in terms of their genetic architecture. This may be because the effect sizes of each variant (Chesmore *et al*., 2018) differ between depression and Neu, which includes the possibility that some variants affect one trait but not the other. A third (non-exclusive) possibility is that Neu or one of its facets is directly related to only a subtype of depression and that other subtypes will have distinct genetic aetiologies (Kendler *et al*., 2013; Hill *et al*., 2019).

In the current study we sought to compare genome-wide association summary statistics for depression and Neu to test whether there are specific genetic contributions to depression that are independent of Neu. First, we sought to identify loci that conferred a higher risk of depression, but not higher Neu, and vice versa. We then annotated the function of any associated loci. Second, we sought to determine whether depression and Neu have any unique genetic correlations with other traits and disorders after adjustment for the other variable.

## Methods

### Data sources

We used depression summary statistics from the Psychiatric Genomics Consortium (PGC) (Wray *et al*., 2018), from the UK Biobank (Howard *et al*., 2019) and from the 23andMe cohort (Hyde *et al*., 2016). We also obtained PGC MD summary statistics that included the 23andMe cohort, but excluded the UK Biobank cohort (*N* = 431 394, 116 404 cases and 314 990 controls, prevalence = 0.27, 9 030 847 variants). We compared the depression summary statistics with summary statistics for Neu (Luciano *et al*., 2017) in UK Biobank (*N* = 329 821, 18 485 883 variants).

### Pairwise GWAS of depression and neuroticism

To identify loci that contribute to variation in liability to depression, but not Neu, we used pairwise GWAS (Pickrell *et al*., 2016) to jointly analyse summary statistics from depression and Neu. Because this method requires that summary statistic sample overlap be minimised, we examined recent meta-analyses (Nagel *et al*., 2018; Howard *et al*., 2019) and identified sources of summary statistics that could yield the largest, independent sample sizes for both sets of summary statistics. We used depression summary statistics from the PGC MDD2 meta-analysis (Wray *et al*., 2018) of PGC and 23andMe data that excluded summary statistics from the UK Biobank sample (*N* cases = 116 404; *N* controls = 314 990); for Neu we used summary statistics from the UK Biobank sample (Luciano *et al*., 2017) (*N* = 329 821). We used the munge_sumstats.py tool (Bulik-Sullivan *et al*., 2015) to convert the summary statistics to *z*-scores and to align and filter effect alleles to a common reference (HapMap 3 SNPs). Using these filtered summary statistics, the *gwas-pw* program (Pickrell *et al*., 2016) models the probability that each locus is associated with only one of the two traits, the probability that the locus has a shared association with both traits, and the probability that each of the genomic regions contains separate loci that are associated with each trait. We analysed the summary statistics by splitting them into genomic segments (1702 in total) that were approximately independent based on linkage disequilibrium (Berisa and Pickrell, 2016). To correct for potential cohort overlap, we first identified loci that were not associated with both traits using the *fgwas* program (Pickrell, 2014). We then retained segments that had a posterior probability of association <0.2 with either trait. Using these non-associated segments, we calculated the correlation of effect sizes across the depression and Neu summary statistics, and then supplied this correlation coefficient to gwas-pw. The gwas-pw program performs a Bayesian test on each genomic region to estimate the probability of unique or overlapping association signals from the two sets of GWAS summary statistics (Pickrell *et al*., 2016).

Using the gwas-pw output, we categorised each segment as being associated with depression only, Neu only, both traits or neither trait. We defined depression-only segments as those that had the highest posterior probability associated with only depression and that also had a genome-wide significant hit in the original GWAS but did not contain a genome-wide significant hit for Neu (and vice versa for Neu-only segments). We defined segments that were associated with neither trait as those that had a total posterior probability of association <0.2 and that did not contain any genome-wide hits for either trait. To examine the depression-only signal, we excluded segments associated with Neu or with both traits from the depression summary statistics, clumped single-nucleotide polymorphisms (SNPs) that were in linkage disequilibrium (LD) (*r*^2^ > 0.1) and within 3 Mb, then used MAGMA (de Leeuw *et al*., 2015) to identify significantly associated (*p* < 2.77 × 10^−6^) genes and conducted GWAS catalogue lookups using FUMA (Watanabe *et al*., 2017). We then conducted the same set of analyses on the Neu-only segments from the Neu summary statistics and the segments associated with both depression and Neu from the depression summary statistics.

### Genetic correlations with depression adjusted for neuroticism

We used cross-trait genetic correlations to identify traits that were related to MD after removing their shared genetic effects with Neu. To start, we first obtained estimates of genetic correlations from GWAS summary statistics that were downloaded from the LD Hub (Zheng *et al*., 2017). The LD Hub results contained information on genetic correlations with MDD from the PGC (Major Depressive Disorder Working Group of the Psychiatric GWAS Consortium, 2013) and Neu from the Genetics of Personality Consortium (GPC) (de Moor *et al*., 2012). We selected 18 traits of interest that were nominally genetically correlated (*p* < 0.01) with either MDD or Neu. These included psychiatric disorders, personality traits, cardiovascular and inflammatory diseases, anthropometric and life-history traits, education and lifestyle factors. We supplemented this list of traits with BMI since obesity has been identified as a potential stratifying factor for depression (Milaneschi *et al*., 2017) and retained the summary statistics for GPC Neu as a check on the method. We also substituted summary statistics from larger and more recent GWAS than those listed in the LD Hub download, where available (online Supplementary Table S1). For each trait of interest, we calculated its genetic correlation using Linkage Disequilibrium Score (LDSC) regression (Bulik-Sullivan *et al*., 2015) with the recent GWAS summary statistics for MD and Neu. For depression we used a meta-analysis of PGC, 23andMe and UK Biobank (Howard *et al*., 2019) and for Neu we used data from UK Biobank (Luciano *et al*., 2017).

Our goal was to determine whether depression's genetic correlation with each trait of interest is (1) explained by the genetic architecture shared between depression and Neu (‘neurotic depression’) or (2) specific to depression and independent of Neu (‘non-neurotic depression’) (and vice versa for ‘depressive neuroticism’ and ‘non-depressive neuroticism’). We estimated the genetic covariance among MD, Neu and each trait using GenomicSEM (Grotzinger *et al*., 2019) and fitted a multiple regression model:
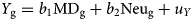
where *Y*_g_, MD_g_ and Neu_g_ are the LDSC genetic values for trait *Y*, major depression and Neu, respectively; *b*_1_ and *b*_2_ are regression coefficients and *u*_*Y*_ is an error term. We used standardised estimates of the regression coefficients to obtain the partial correlations of MD after removing shared overlap with Neu, and vice versa. We first identified traits that were genetically correlated with MD but not with Neu. We then identified traits that were genetically correlated with both depression and Neu but that had a substantial partial genetic correlation with depression after adjusting for Neu (MD·adjNeu) but not with Neu after adjusting for depression (Neu·adjMD). Finally, we identified traits with the opposite patterns of having genetic correlations that were specific to Neu or to Neu after adjusting for depression. We tested whether unadjusted and adjusted correlations were different from zero and whether adjusted correlations were smaller than their unadjusted counterparts and corrected for multiple testing using False Discovery Rate (Benjamini and Yekutieli, 2001).

## Results

### Pairwise GWAS of depression and neuroticism

**Fig. 1.**
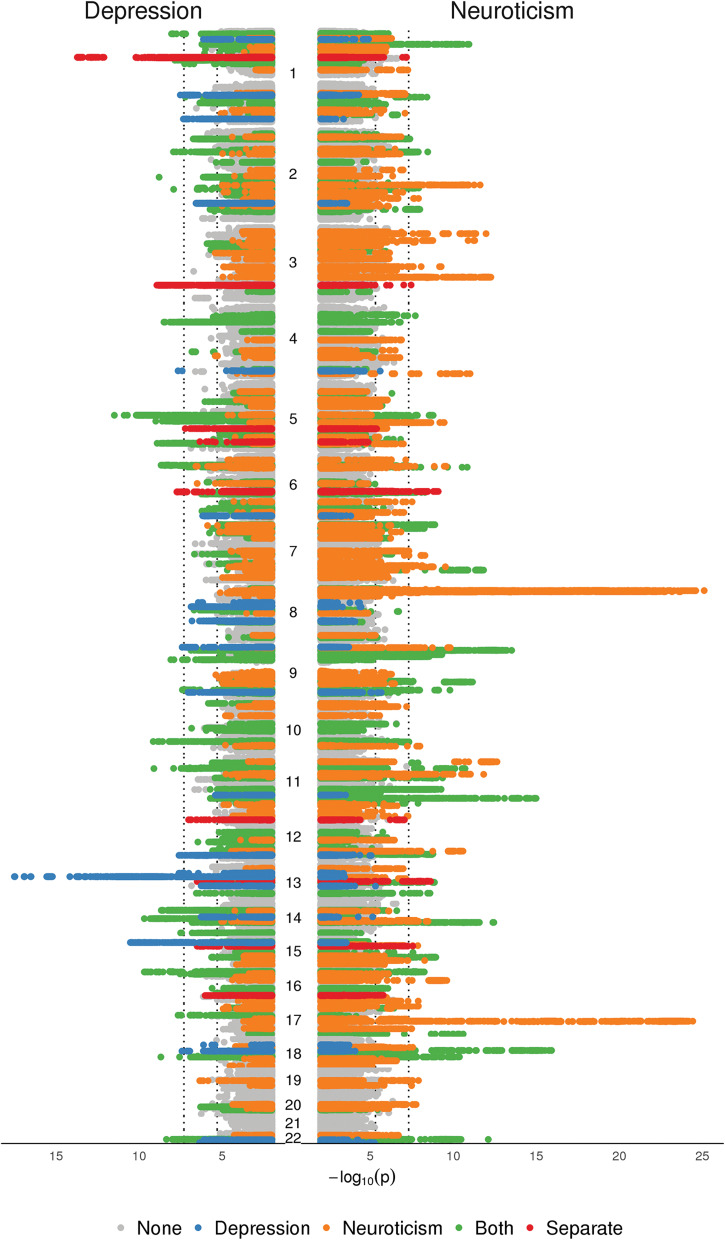
Manhattan plot of pairwise GWAS of depression and Neu with genomic segments partition by association with depression-only (blue), Neu only (orange), both traits (green), separate associations (red) or neither trait (grey).

We used pairwise GWAS (Pickrell *et al*., 2016) between depression and Neu to partition genomic segments with association signals for MD only, for Neu only, for both traits or that contained different associations for each trait. These associations were assessed by posterior probabilities, where higher values mean more probable. The correlation in beta coefficients from the summary statistics in genomic segments that were not associated with either MD or Neu was *r* = 0.005, suggesting there was no undetected sample overlap between the two studies. We identified nine genomic segments containing loci that influence depression but do not associate with Neu (i.e. association with depression-only had the highest posterior probability) (Fig. 1, Table 1). This represented 24% of the total 37 genomic segments that contained loci previously associated with depression. The analysis indicated that there were three genomic segments that contained separate associations for depression and Neu (online Supplementary Table S2 and Figs S1–S3). There were 25 GWAS hits for depression that were also associated with Neu; in addition, there were 45 more regions that, while below the threshold of genome-wide significance for depression, the pairwise analysis indicated were associated with both depression and Neu. Finally, there were also 40 genomic segments that were significantly associated only with Neu (online Supplementary Table S2).

**Table 1.** Genomic regions that are likely associated with depression but not with Neu that also contain a genome-wide significant SNP for depression

Chr	Region (Mb)	Prob(Dep only)	Prob(N only)	Prob(both)	Prob(separate)	Top SNP	BP	A1/2	Dep. GWAS *p*-value
1	175.090–177.433	0.533	0.001	0.287	0.094	rs10913112	175 913 828	T/C	3.02 × 10^−8^
1	241.583–242.071	0.657	3 × 10^−4^	0.176	0.032	rs4660091	241 628 430	T/C	4.64 × 10^−8^
4	174.267–176.568	0.011	0.004	0.004	0.001	rs75990749	175 349 867	C/G	2.22 × 10^−8^
9	1.920–3.185	0.449	0.001	0.413	0.053	rs7036618	2 990 717	A/C	3.92 × 10^−8^
12	119.759–121.989	0.380	0.005	0.222	0.092	rs77741769	121 363 835	T/C	2.53 × 10^−8^
13	43.109–44.901	0.595	0.001	0.151	0.123	rs4143229	44 327 799	A/C	2.50 × 10^−8^
13	53.340–54.681	0.800	3 × 10^−13^	0.108	0.120	rs1343605	53 647 048	A/C	3.09 × 10^−18^
15	37.461–38.528	0.514	7 × 10^−7^	0.363	0.001	rs834629	37 678 862	T/C	2.97 × 10^−11^
18	36.366–37.683	0.337	0.004	0.285	0.046	chr18_36904688_I	36 904 688	D/I	3.80 × 10^−8^

For each segment, the posterior probability of association (higher value means more probable) with depression only [Prob(Dep only)] is compared with Neu only [Prob(N only)], with the same association signal for both traits [Prob(both)] or with separate association signals effecting each trait [Prob(separate)]. For each region, the top SNP associated with MDD is listed along with its base pair position (BP) and GWAS *p*-value.

We used MAGMA (de Leeuw *et al*., 2015) to identify genes associated (*p* < 2.77 × 10^−6^) with the partitioned genomic segments. There were 30 genes significantly associated with depression only (online Supplementary Table S3), 203 genes associated with Neu only (online Supplementary Table S4) and 104 genes associated with both depression and Neu (online Supplementary Table S5). We used FUMA (Watanabe *et al*., 2017) to conduct GWAS catalogue lookups on these gene sets (online Supplementary Table S6). MD was uniquely associated with gene sets that are linked to N-glycan levels (*p* = 1.1 × 10^−8^) and to coronary heart disease (*p* = 3.2 × 10^−3^). Neu-only gene sets were related to traits such as intracranial volume, Parkinson's disease and high-density lipoprotein cholesterol. The gene sets shared by depression and Neu were related to cross-disorder psychiatric traits, coffee consumption and epilepsy, among other traits (online Supplementary S6).

### Partial genetic correlations with depression removing neuroticism

**Fig. 2.**
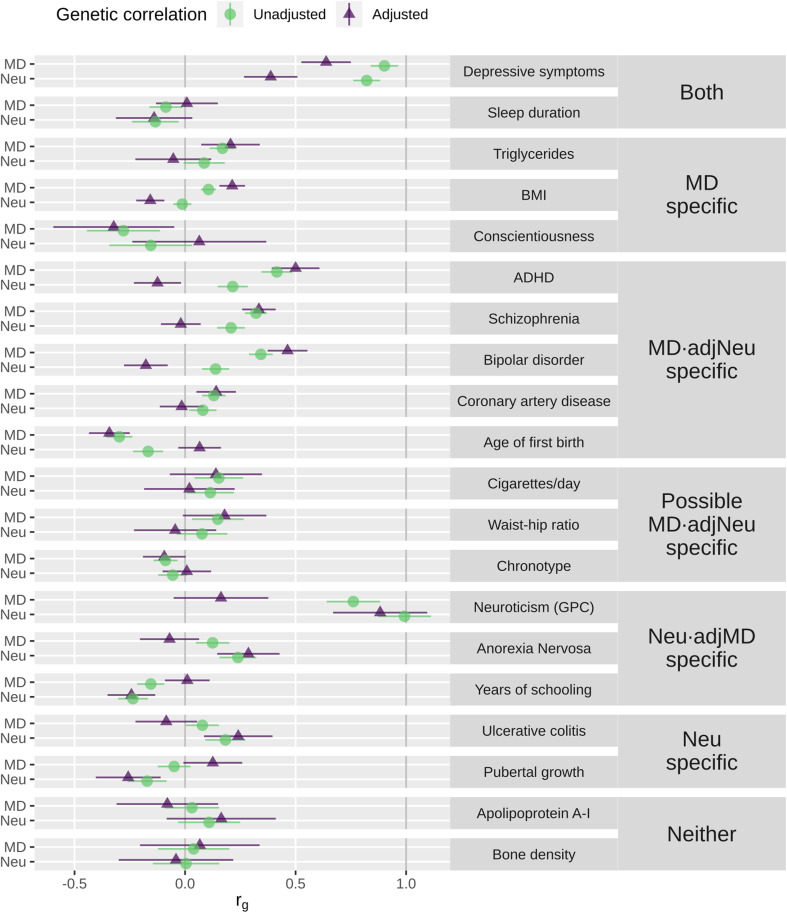
Genetic correlations of traits with MD and with Neu. Full, unadjusted correlations (green circles) and partial genetic correlations after adjusting for the other variable (purple triangles) with 95% confidence intervals. We grouped traits based on the patterning of full and partial genetic correlations: both = genetically correlated with MD and Neu, with specific or entirely shared overlap; MD specific = genetically correlated with depression but not Neu; MD·adjNeu specific = fully correlated with both MD and Neu, but partially correlated with only MD; Possible MD·adjNeu specific = fully or partially correlated with depression and not Neu, but substantial overlap in effect sizes; Neu·adjMD specific: fully correlated with both MD and Neu, but partially correlated with only Neu; Neu specific = genetically correlated with Neu but not depression; neither = not genetically correlated with either depression or Neu.

The LD score genetic correlation between MD and Neu was 0.680 ± 0.028 s.e. We fitted multiple regression models in GenomicSEM to estimate the unadjusted genetic correlation of each trait of interest with MD and with Neu and the partial genetic correlation with depression where the shared overlap with Neu has been removed (MD·adjNeu) and with Neu where overlap with depression has been removed (Neu·adjMD) (Fig. 2, online Supplementary Table S1). We grouped traits based on the pattern of their unadjusted and adjusted genetic correlations with MD and Neu. Two traits were genetically correlated with both MD and Neu that were slightly (depressive symptoms) or completely (sleep duration) attenuated when adjusting for the other variable. Three traits (triglycerides, BMI and conscientiousness) were genetically correlated with MD but not Neu while another two traits (ulcerative colitis and pubertal growth) were genetically correlated with Neu but not with depression. There are two traits (apolipoprotein A-1 and bone density) that were correlated with neither MD nor Neu.

The multiple regression analyses identified traits correlated with MD and Neu but that had specific genetic correlations with only one of these phenotypes after adjusting for shared overlap with the other, wherein the partial correlation was maintained for the focal phenotype but went to zero or had the opposite sign for the other phenotype. MD had specific genetic correlations with attention deficit hyperactivity disorder (ADHD), schizophrenia, bipolar disorder, coronary artery disease and age of first birth. There were three additional traits [cigarettes smoked per day, waist–hip ratio and chronotype (‘morningness’)] that showed a similar pattern of full and partial correlations with MD, but where the effect sizes overlapped with those for Neu, and thus their status of having a specific relationship was less well supported. Finally, there were three traits (Neu measured in the GPC, anorexia and years of schooling) that showed specific relationships with Neu after removing shared overlap with MD.

## Discussion

We conducted complementary analyses to separate the specific genetic features of MD from those that overlap with Neu and vice versa. The pairwise GWAS analysis using depression results from the PGC and 23andMe and Neu results from UK Biobank revealed nine genomic regions that were significantly associated with depression but not with Neu. Several of the associated regions contained genes of known function: rs10913112 is downstream of *RFWD2* (ring finger and WD repeat domain 2), a gene that can promote tumour growth (Dornan *et al*., 2004); rs4660091 is near the fumarate hydratase gene (*FH*) which is involved in the Krebs cycle; rs4143229 is in an intron of the ecto-NOX disulphide-thiol exchanger 1 gene (*ENOX1*) which is expressed in the nervous systems and has been implicated in autoimmune disorders (Landouré *et al*., 2012) and rs1343605 is near *OLFM4*, a gene that has been linked to depression (Wray *et al*., 2018), inflammation and cancer (Liu and Rodgers, 2016).

There were 40 independent regions that were associated with Neu but not depression. These included the two large inversion polymorphisms, located on chromosomes 8 and 17, that have previously been associated with Neu (Okbay *et al*., 2016; Luciano *et al*., 2017). These polymorphisms are thought to influence the regulation of the genes in these regions, some of which are highly expressed in the nervous (*MAPT*, *MSRA*, *MTMR9*) and immune (*BLK*, *MFHAS1*) systems (Okbay *et al*., 2016). The remaining regions of interest were associated with both depression and Neu. These included the MHC region; *DRD2*, the dopamine D2 receptor involved in mood and emotion; *MEF2C*, a gene that regulates synaptic function; *TCF4*, a regulator of prefrontal neuronal excitability and *RBFOX1*, a gene splicing regulator that has also been implicated in other psychiatric disorders (Okbay *et al*., 2016; Wray *et al*., 2018; Howard *et al*., 2019). There were 10 regions that did not contain genome-wide significant associations in either depression or Neu summary statistics but that, when analysed jointly in the pairwise analysis, appeared to be associated with both traits. The novel loci included SNPs in or near the *DENND1B* (DENN domain containing 1B) gene, which has been implicated in childhood asthma (Sleiman *et al*., 2010); RNF103 (ring finger protein 103), a suspected antidepressant target (Yamada *et al*., 2000) and genes related to axon guidance (DCC netrin 1 receptor) (Kolodziej *et al*., 1996). There was also a non-novel locus in the PCLO (piccolo presynaptic cytomatrix protein) gene involved in synaptic vesicle trafficking that, while not genome-wide significant in the PGC and 23andMe meta-analysis (Wray *et al*., 2018) did reach genome-wide significance separately for MDD in single cohort (Mbarek *et al*., 2017) that is included in the PGC meta-analyses.

The pairwise GWAS also suggested three regions that contained separate association signals for depression and Neu. For example, a region (71.6–74.3 Mb on chromosome 1, online Supplementary Fig. S1) contained two association signals for depression. The first signal (rs1460942) was shared with Neu and was close to the neuronal growth regulator 1 (*NEGR1*) gene. A second association signal (rs12129573) was in the intron of an uncharacterised non-protein coding RNA (*LOC105378800*). In a further region, a signal was found (97.8–100.6 Mb on chromosome 6) that showed a clear separation between the association signals for each trait (online Supplementary Fig. S3). The SNP associated with depression (rs12202410) was near the F-box and leucine-rich repeat protein 4 (*FBXL4*) gene which is related to energy homoeostasis (Gai *et al*., 2013).

Using a GWAS catalogue lookup with FUMA (Watanabe *et al*., 2017) on the pairwise GWAS results, we found that genes associated with depression, but not with Neu, were also associated with glycosylation and coronary heart disease. This suggests that there may be subtypes of depression involving inflammation and cardiovascular disease that are separate from subtypes of depression associated with Neu. In contrast, genes that were associated with both depression and Neu were also associated with psychological, behavioural and psychiatric traits such as schizophrenia, autism spectrum disorder and intelligence, suggesting that they may influence behaviour and cognitive function more generally.

Differential association of MD with other traits, once shared overlap with Neu was accounted for, was also shown in our analysis of LD score genetic correlations. We found that, unlike Neu, MD was genetically correlated with BMI, triglyceride levels and coronary artery disease, suggesting the atypical depressive subtype related to cardio-metabolic traits (Lasserre *et al*., 2016; Milaneschi *et al*., 2017). Even after removing shared genetic overlap with Neu, MD was still specifically related to ADHD, schizophrenia, bipolar disorder, coronary artery disease and age of first birth; and also possibly to smoking, waist–hip ratio and chronotype (‘morningness’). The robust association with schizophrenia and bipolar disorder after adjusting for overlap with Neu may indicate genetic subtype heterogeneity among depression cases or the over-inclusiveness of minimal depression phenotyping (Cai *et al*., 2019). In contrast, the genetic architecture of depression that is shared with Neu explained all or most of the genetic correlation of depression with anorexia and years of schooling, which arise from the worry/vulnerability and anxiety/tension subfactors of Neu (Hill *et al*., 2019).

One limitation of our study is that by using summary statistics from GWAS, we were only able to assess the overlap in genetic architecture between depression and Neu that arises from common variants. Even biobank-sized samples of millions of participants can be underpowered for detecting associations with rare variants unless such variants have very large effect sizes (Visscher *et al*., 2017). While common variants contribute a large proportion to phenotype heritabilities (Shi *et al*., 2016), and thus trait covariances, it is not known how the strength of any correlation will differ between rare and common polymorphisms (Shi *et al*., 2016, 2017). Furthermore there is evidence that rare variants are associated with both Neu and depression (Zeng *et al*., 2016; Hill *et al*., 2018). Much will depend on the evolutionary history of both phenotypes. If, for example, antagonistic pleiotropy, where a variant has opposite effects on two phenotypes, is maintaining common variants, then rare variants might be more likely to affect both traits in the same direction (Carter and Nguyen, 2011). A second limitation is that we used summary statistics for MD that were meta-analysed from studies with varying levels of phenotyping depth and thus may be picking up on genetic overlap that is more broadly shared with other psychiatric disorders rather than specific to clinically defined MDD (Cai *et al*., 2019). Another limitation is that our source studies used case/control definitions of depression and sum-score quantities of Neu, while other analyses of individual depressive symptoms (Thorp *et al*., 2019) and factors of Neu (Hill *et al*., 2019) shows that depression and Neu are composed of subfactors that themselves have unique associations with other traits.

Neu is a major risk factor for depression (Bagby *et al*., 1995; Kendler *et al*., 2004), the two traits are strongly genetically correlated (Jardine *et al*., 1984; Fanous *et al*., 2002; Luciano *et al*., 2017), and Mendelian randomisation analysis has pinpointed high Neu as a cause of depression (Howard *et al*., 2019). Our results confirm that the majority of specific genetic variants associated with depression are shared with Neu. However, we also identified several association signals and overlap with other traits that were unique to depression. In particular, the pairwise GWAS analysis both identified new associations with depression and separated out associations that were specific to depression while the partial genetic correlation analysis identified other phenotypes that are related to depression even after removing shared genetic overlap with Neu. This suggests that that depression and Neu are not *just* noisy measures of the same underlying liability. If these differences represent distinct genetic subtypes of depression, then most cases of depression will stem from this Neu–depression nexus, while a smaller proportion may have aetiologies that are distinct from Neu. Some of these associations, such as between depression, chronotype and metabolic phenotypes, are suggestive of endogenous depression's features but do not point to all the characteristics of this previously described subtype. Confirming known depression subtypes and identifying new subtypes will be useful for phenotypic and clinical stratification. The unique associations of triglyceride levels and BMI with depression, but not at all with Neu, confirms that depression with comorbid obesity and other metabolic factors should be studied as a subtype when exploring aetiology and testing treatment efficacy. The partial genetic correlations that ADHD, schizophrenia and bipolar disorder have with depression after adjusting for Neu imply that polygenic risk scores for these other disorders may be useful to screen or stratify participants even if they do not manifest these other disorders.

Neither depression (Kendler *et al*., 2013; Thorp *et al*., 2019) nor Neu (Hill *et al*., 2019) is completely genetically homogeneous. Because the common variant genetic overlap is high between different forms of assessing depression (clinically ascertained, brief questionnaire, hospital records) (Howard *et al*., 2018; Wray *et al*., 2018), our results suggest that much of the shared genetic variance, where depression and non-depression are on a continuum, is wrapped-up in the Neu–depression nexus, and that going forward additional studies based on a more refined, symptom-level analysis may be more revealing for non-Neu-related forms of depression.

## Supplementary material

For supplementary material accompanying this paper visit https://doi.org/10.1017/S0033291719002629. click here to view supplementary material

## Data

The full MDD summary statistics from Hyde *et al*. are made available through 23andMe to qualified researchers under an agreement with 23andMe that protects the privacy of the 23andMe participants. Visit https://research.23andme.com/collaborate for more information and to apply to access the data. Summary statistics from the Psychiatric Genomics Consortium (Wray *et al*., 2018; Howard *et al*., 2019) are available for download from http://www.med.unc.edu/pgc/. Summary statistics for Neu in UK Biobank are available from the Centre for Cognitive Aging and Cognitive Epidemiology http://www.ccace.ed.ac.uk/node/335.
